# Supplementary figures and images for: Sex-specific distribution and classification of Wolbachia infections and mitochondrial DNA haplogroups in Aedes albopictus from the Indo-Pacific
Source: PLoS Negl Trop Dis. 2022 Apr 13;16(4):e0010139. doi: 10.1371/journal.pntd.0010139 (PMC9037918; doi:10.1371/journal.pntd.0010139)

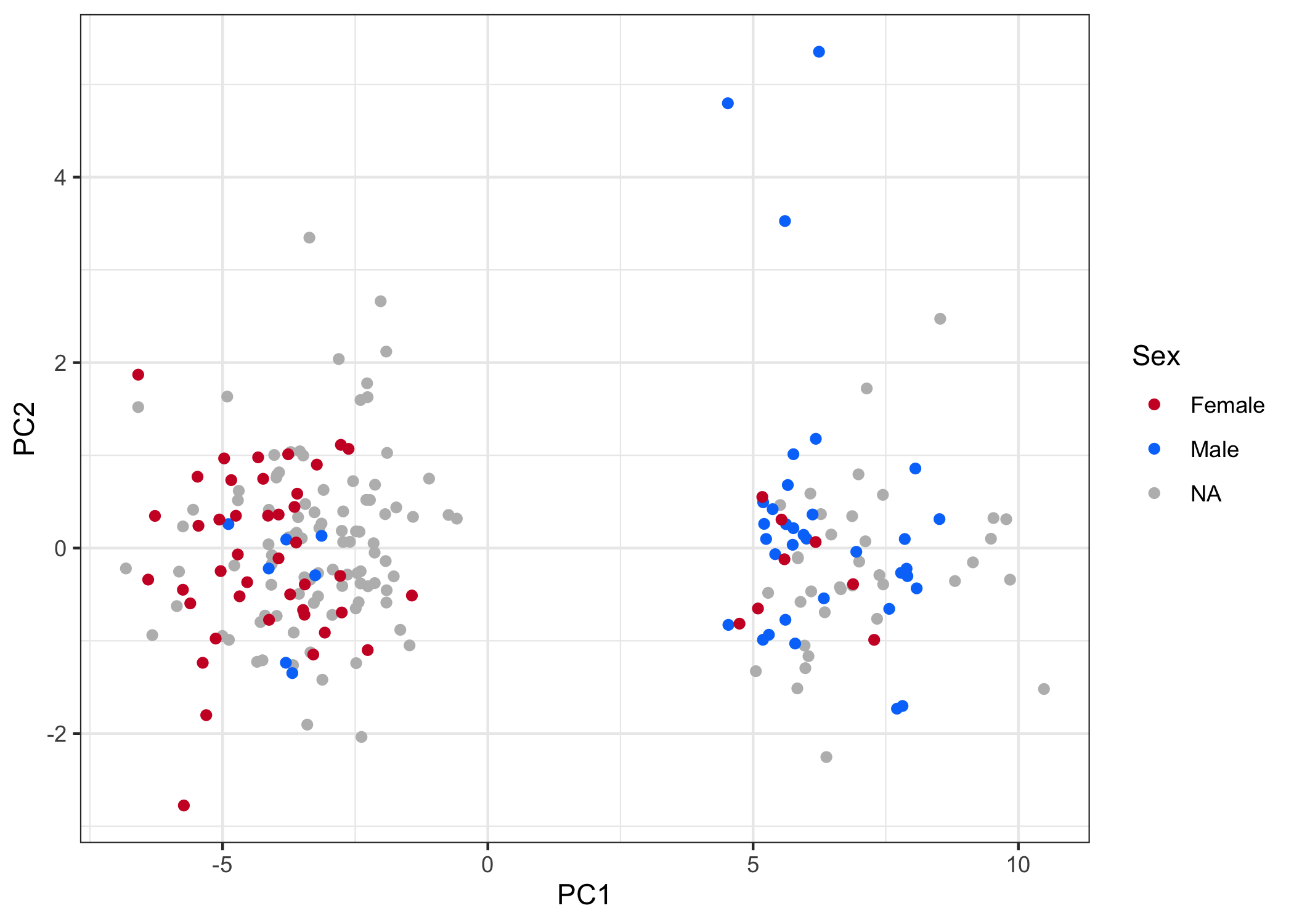

Supplement: S1 Fig — (TIF) [file pntd.0010139.s001.tif]

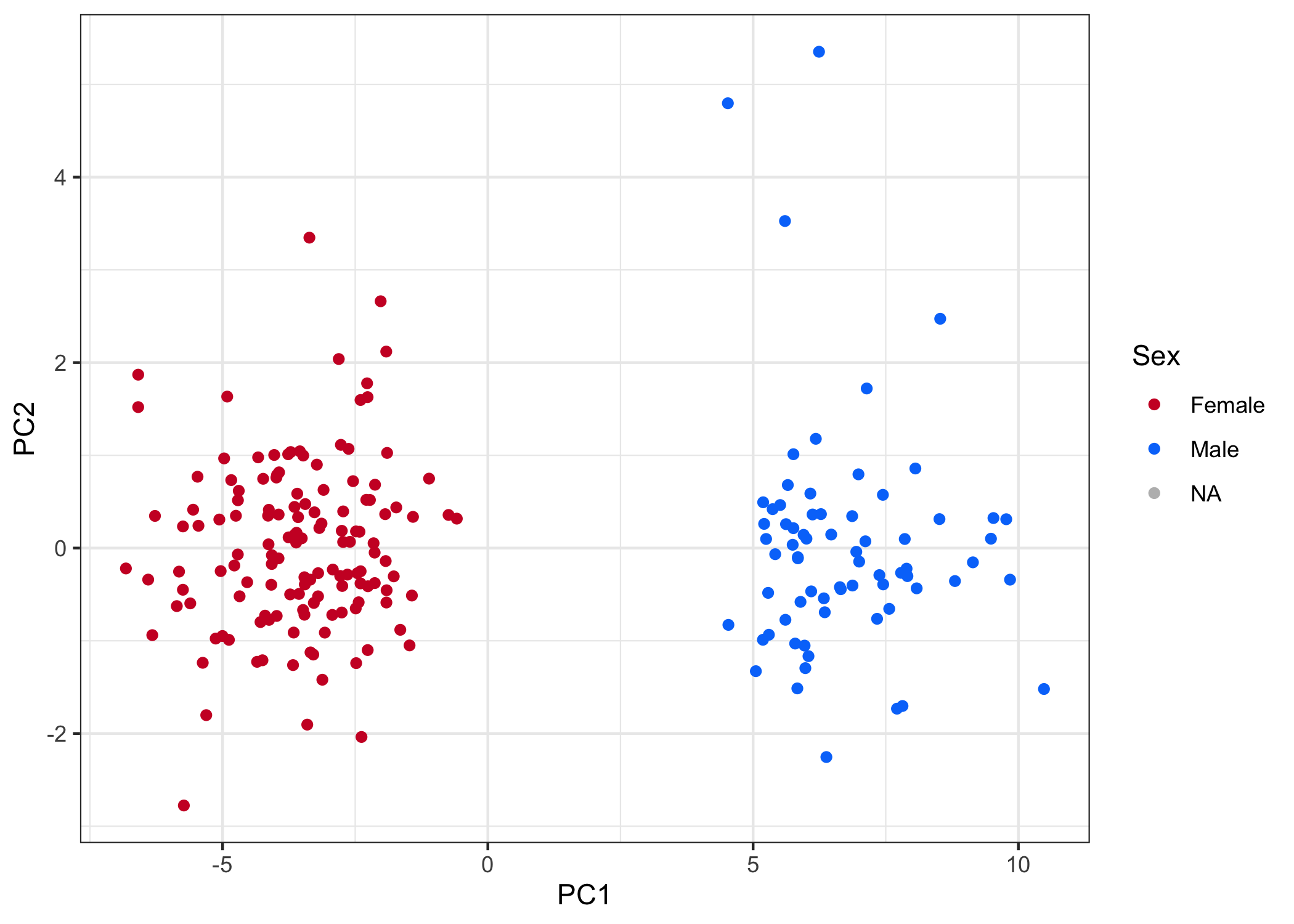

Supplement: S2 Fig — (TIF) [file pntd.0010139.s002.tif]
