# Supplementary material for: Sex-specific distribution and classification of Wolbachia infections and mitochondrial DNA haplogroups in Aedes albopictus from the Indo-Pacific
Source: PLoS Negl Trop Dis. 2022 Apr 13;16(4):e0010139. doi: 10.1371/journal.pntd.0010139 (PMC9037918; doi:10.1371/journal.pntd.0010139)
Supplement: S2 Table — (DOCX) [file pntd.0010139.s004.docx]

Table S2. Field surveys of *Wolbachia* infections in populations of *Aedes albopictus* from previous studies.

|  | **Sex** | **Supergroup** | | | | **Reference** |
| --- | --- | --- | --- | --- | --- | --- |
|  |  | *w*AlbA (%) | *w*AlbB (%) | *w*AlbA+ *w*AlbB (%) | Uninfected (%) |  |
| Malaysia (M, S, T, Pe, Pa)* | Male | 0 | 46.1(47/102) | 49.0(50/102) | 4.9(5/102) | [1] |
|  | Female | 0.7(1/142) | 0.7(1/142) | 97.2(138/142) | 1.4(2/142) |  |
| Malaysia (M, S, T, KI, CI)* | Male | 0 | 67.4(31/46) | 32.6(15/46) | 0 | [2] |
|  | Female | 0 | 0 | 100.0 (58/58) | 0 |  |
| Malaysia (Pe, NS, Sa, WP, KL, KV)* | Male | 0 | 6.8(5/74) | 87.8(65/74) | 5.4  (4/74) | [3] |
|  | Female | 1.4(3/212) | 0.5(1/212) | 92.9(197/212) | 5.2  (11/212) |  |
| Malaysia (S, KL, Pa, Pe, Ke, T)* | Unspecific | 14.3(21/147) | 19.0 (28/147) | 66.7(98/147) | 0 | [4] |
| Korea | Female | 2.0(15/739) | 0 | 98.0(724/739) | 0 | [5] |
| Russia | Unspecific | 1.0(4/411) | 44.8(184/411) | 11.2(46/411) | 43.1(177/411) | [6] |
| La Reunion Island, Madagascar | Male | 0 | 54.8(23/42) | 45.2(19/42) | 0 | [7] |
|  | Female | 0 | 0 | 100(42/42) | 0 |  |
| China (Guiyang) | Female | 0 | 15.0(9/60) | 35.0(21/60) | 50.0(30/60) | [8] |
| China (Guangzhou) | Male | 8.0(9/113) | 0 | 92.0(104/113) | 0 | [9] |
|  | Female | 0 | 0.9(1/110) | 99.1(109/110) | 0 |  |
| China (14 populations) | Female | 9.3 (12/129) | 10.1(13/129) | 70.5(91/129) | 10.1(13/129) | [10] |
| Argentina | Male | 0 | 12.5(1/8) | 62.5(5/8) | 25.0(2/8) | [11] |
|  | Female | 0 | 16.7(3/18) | 66.7(12/18) | 16.7(3/18) |  |
| 18 populations around the world | Female | 0 | 0 | 100.0 (179/179) | 0 | [12] |
| India | Male | 4.2(29/689) | 0 | 94.6(652/689) | 1.2(8/689) | [13] |
|  | Female | 2(12/602) | 0 | 98(588/602) | 0.3(2/602) |  |
| Madagascar | Male | 0 | 2.2(6/278) | 97.8(272/278) | 0 | [14] |
|  | Female | 0 | 1.0(4/417) | 99.0(413/417) | 0 |  |
| Texas, Florida and New Mexico | Male | 2.7(1/37) | 27.0(10/37) | 70.3(26/37) | 0 | [15] |
|  | Female | 2.8(1/36) | 2.8(1/36) | 94.4(34/36) | 0 |  |
| Brazil | Male | 0 | 0 | 100(58/58) | 0 | [16] |
|  | Female | 0 | 1.2(1/98) | 98.8(83/84) | 0 |  |
| Thailand | Unspecific | 0.9(3/320) | 1.6(5/320) | 97.5(312/320) | - | [17] |
| Total |  | 2.2(111/5153) | 7.3(374/5153) | 85.6(4411/5153) | 5.0(257/5153) | - |

*Abbreviation: M: Malacca, S: Selangor, T: Terengganu, Pe: Perak, Pa: Pahang, KI: Ketam Island, CI: Carey Island, NS: Negeri Sembilan, Sa: Sabah, WP: Wilayah Persekutuan, KV: Klang Valley, KL: Kuala Lumpur, Ke: Kelantan.

Reference

1. Ahmad NA, Vythilingam I, Lim YAL, Zabari N, Lee HL. Detection of *Wolbachia* in *Aedes albopictus* and their effects on Chikungunya virus. Am J Trop Med Hyg. 2017;96(1):148-56. Epub 2016/12/07. doi: 10.4269/ajtmh.16-0516. PubMed PMID: 27920393; PubMed Central PMCID: PMCPMC5239683.

2. Afizah AN, Mahirah MN, Azahari AH, Asuad MK, Nazni WA, Lee HL. Absence of *Aedes aegypti* (L.) on an ecological island: Competitive exclusion? Southeast Asian J Trop Med Public Health. 2015;46(5):850-6. Epub 2016/02/13. PubMed PMID: 26863856.

3. Joanne S, Vythilingam I, Yugavathy N, Leong CS, Wong ML, AbuBakar S. Distribution and dynamics of *Wolbachia* infection in Malaysian *Aedes albopictus*. Acta Trop. 2015;148:38-45. Epub 2015/04/23. doi: 10.1016/j.actatropica.2015.04.003. PubMed PMID: 25899523.

4. Noor-Shazleen-Husnie MM, Emelia O, Ahmad-Firdaus MS, Zainol-Ariffin P, Aishah-Hani A. Detection of *Wolbachia* in wild mosquito populations from selected areas in Peninsular Malaysia by loop-mediated isothermal amplification (LAMP) technique. Trop Biomed. 2018;35(2):330-46. Epub 2018/06/01. PubMed PMID: 33601807.

5. Park CH, Lim H, Kim H, Lee WG, Roh JY, Park MY, et al. High prevalence of *Wolbachia* infection in Korean populations of *Aedes albopictus* (Diptera: Culicidae). J Asia-Pacific Entomol. 2016;19:191-4.

6. Shaikevich EV, Patraman IV, Bogacheva AS, Rakova VM, Zelya OP, Ganushkina LA. Invasive mosquito species Aedes albopictus and *Aedes aegypti* on the Black Sea coast of the Caucasus: genetics (COI, ITS2), *Wolbachia* and *Dirofilaria i*nfections. Vavilov J Genet Breeding. 2018;22(5):574-85. doi: 10.18699/VJ18.397.

7. Tortosa P, Charlat S, Labbe P, Dehecq JS, Barre H, Weill M. *Wolbach*ia age-sex-specific density in *Aedes albopictus*: a host evolutionary response to cytoplasmic incompatibility? PLoS One. 2010;5(3):e9700. Epub 2010/03/20. doi: 10.1371/journal.pone.0009700. PubMed PMID: 20300514; PubMed Central PMCID: PMCPMC2838780.

8. Wu YN, Wen S, Liang Qg, Yang X, Wu JH, Zhu RF, et al. The Preliminary Investigation on Symbiotic Bacteria (*Wolbachia*) of Common Mosquito in Guiyang. J Guizhou Med Uni. 2017;10:1130-3.

9. Zhang D, Zhan X, Wu X, Yang X, Liang G, Zheng Z, et al. A field survey for *Wolbchia* and phage WO infections of *Aedes albopictus* in Guangzhou City, China. Parasitol Res. 2014;113(1):399-404. Epub 2013/11/14. doi: 10.1007/s00436-013-3668-9. PubMed PMID: 24221888.

10. Guo Y, Song Z, Luo L, Wang Q, Zhou G, Yang D, et al. Molecular evidence for new sympatric cryptic species of *Aedes albopictus* (Diptera: Culicidae) in China: A new threat from *Aedes albopictus* subgroup? Parasit Vectors. 2018;11(1):228. Epub 2018/04/06. doi: 10.1186/s13071-018-2814-8. PubMed PMID: 29618379; PubMed Central PMCID: PMCPMC5885320.

11. Chuchuy A, Rodriguero MS, Ferrari W, Ciota AT, Kramer LD, Micieli MV. Biological characterization of *Aedes albopictus* (Diptera: Culicidae) in Argentina: implications for arbovirus transmission. Sci Rep. 2018;8(1):5041. Epub 2018/03/24. doi: 10.1038/s41598-018-23401-7. PubMed PMID: 29568046; PubMed Central PMCID: PMCPMC5864732.

12. Armbruster P, Damsky WE, Jr., Giordano R, Birungi J, Munstermann LE, Conn JE. Infection of New- and Old-World *Aedes albopictus* (Diptera: Culicidae) by the intracellular parasite *Wolbachia*: implications for host mitochondrial DNA evolution. J Med Entomol. 2003;40(3):356-60. Epub 2003/08/29. doi: 10.1603/0022-2585-40.3.356. PubMed PMID: 12943116.

13. Das B, Satapathy T, Kar SK, Hazra RK. Genetic structure and *Wolbachia* genotyping in naturally occurring populations of *Aedes albopictus* across contiguous landscapes of Orissa, India. PLoS One. 2014;9(4):e94094. Epub 2014/04/10. doi: 10.1371/journal.pone.0094094. PubMed PMID: 24714653; PubMed Central PMCID: PMCPMC3979767.

14. Zouache K, Raharimalala FN, Raquin V, Tran-Van V, Raveloson LH, Ravelonandro P, et al. Bacterial diversity of field-caught mosquitoes*, Aedes albopictus* and *Aedes aegypti*, from different geographic regions of Madagascar. FEMS Microbiol Ecol. 2011;75(3):377-89. Epub 2010/12/24. doi: 10.1111/j.1574-6941.2010.01012.x. PubMed PMID: 21175696.

15. Kulkarni A, Yu W, Jiang J, Sanchez C, Karna AK, Martinez KJL, et al. *Wolbachia* pipientis occurs in *Aedes aegypti* populations in New Mexico and Florida, USA. Ecol Evol. 2019;9(10):6148-56. Epub 2019/06/05. doi: 10.1002/ece3.5198. PubMed PMID: 31161026; PubMed Central PMCID: PMCPMC6540660.

16. de Albuquerque AL, Magalhaes T, Ayres CF. High prevalence and lack of diversity of *Wolbachia* pipientis in *Aedes albopictus* populations from Northeast Brazil. Mem Inst Oswaldo Cruz. 2011;106(6):773-6. Epub 2011/10/21. doi: 10.1590/s0074-02762011000600021. PubMed PMID: 22012236.

17. Kittayapong P, Baisley KJ, Sharpe RG, Baimai V, O'Neill SL. Maternal transmission efficiency of *Wolbachia* superinfections in *Aedes albopictus* populations in Thailand. Am J Trop Med Hyg. 2002;66(1):103-7.
