# Supplementary material for: Sex-specific distribution and classification of Wolbachia infections and mitochondrial DNA haplogroups in Aedes albopictus from the Indo-Pacific
Source: PLoS Negl Trop Dis. 2022 Apr 13;16(4):e0010139. doi: 10.1371/journal.pntd.0010139 (PMC9037918; doi:10.1371/journal.pntd.0010139)
Supplement: Attachment — Submitted filename: Revision letter to Eidtor.docx [file pntd.0010139.s006.docx]

Dear Dr Kittayapong and Dr Lambrechts,

We appreciate the constructive feedback from the reviewers. We’ve addressed these points and improved the manuscript and look forward to further evaluation. The following is a point by point response to the reviewer’s feedback.

Sincerely,

Ary Hoffmann and Qiong Yang

**Reviewers' Comments:**

**Methods**

Reviewer 1

1. Mitochondrial COI as a DNA barcode and the trimmed 623 bp sequence was analysed with Geneious 9.18 software (Kearse et al., 2012) to investigate SNP variation among samples. These methods may not be sufficient to identify species.

**Genome sequencing data (ddRAD-seq) obtained from these *Ae. albopictus* samples have been used for population genetic analysis in our previous study, where genotypes were filtered using a bioinformatics pipeline. In the present study, one set of *Ae. albopictus*-specific primers was used to confirm that individual mosquitoes were correctly identified as *Ae. albopictus* and as a qPCR positive control. We have confirmed the *Ae. albopictus* species before we started using COI gene marker to perform the phylogenetic analysis.**

**Previous studies indicated that the molecular marker COI gene is an efficient taxonomic method, which gives an accurate solution to the complex problems of mosquito species identification (Kumar et al., 2007, Kamgang et al., 2011 and Guo et al., 2018). Results of the present investigation also confirmed that trimmed CO1 gene sequences can be successfully used for identification and phylogenetic analysis of *Ae. albopictus* species.**

**We have rewritten the sentence (lines 103-104) to emphasize the previous use of our pipeline for species identification.**

2. ML sex classification with ddRAD-seq ，How reliable is this method?

**To test the performance of the ML sex classification method, we obtained an independent dataset (i.e. samples that the model had not seen before) of ddRAD-seq data from mosquitos that had been morphologically sexed, then compared the model classification to the morphology classifications.**

**We believe the method is reliable as we were able to obtain a high level of accuracy (97.6%) as reported in table 2 and we have reiterated this now at 282-3.**

3. Wolbachia detection via qPCR assay，What are the advantages and confirmations of this method compared with general PCR.

**While the traditional assays are sufficient for small-scale *Wolbachia* screening, the qPCR assays provide high throughput options to detect and quantify *Wolbachia* infection in *Ae. albopictus.* Specially, under field conditions, mosquito specimens often comprise a mixture of *Wolbachia*-infected and uninfected individuals. qPCR is a robust screening assay that can simultaneously detect *Wolbachia* and distinguish between multiple *Wolbachia*** **strains which we have used extensively in our *Aedes* studies. We have added a couple of examples of this use at line 184.**

**Results**

Reviewer 1

1. Figure 6. wAlbA density and wAlbB density in individual females (A-F) and males (G, H) across populations.Why do some have linear correlation and some don't? What is the significance of this difference? Why?

**Regression lines are only included where significant linear associations were detected as previously noted in the legend. Associations between densities of the two infections were always positive and significant (P < 0.05) in females. We found no associations between the density of the infections in males, so males did not have regression lines.**

**Our data show that (Fig 4), for wAlbA density, there was a significant effect of sex and population on density when considering only superinfected individuals. Previous studies (Tortosa et al., 2010) also showed that the wAlbA density strongly decreases with age, towards complete loss in *Ae. albopictus* males collected in the field. We believe this is why there was a consistent positive association of these infections in females from all samples and an absence of any pattern in males. Our results also highlight the absence of antagonistic interactions among the infections. We have added some information to the legend of Figure 6 to indicate why some have regression lines.**

2. Test set confusion matrix for sex prediction of mosquitoes based on SNP markers identified from a Torres Strait dataset but tested against sexed samples from other populations. How accurate is the prediction accuracy of SNP markers?

**The sex prediction model was based on sequencing depth of certain ddRAD tags from ddRAD sequencing, therefore, we expected performance to not differ much between populations, unlike they would if we had used SNPs. We have clarified in the revision that the model uses sequencing depth as the model features (lines 32, 87–88, 104, 291, 521, 532) rather than the genotype of the SNPs themselves, and thank the reviewer for identifying this point of ambiguity.**

**Regarding prediction accuracy, model validation using an independent test set obtained an accuracy of 97.6% (table 2) as now further emphasized (see above response).**

**Summary and General Comments**

Reviewer 2

1. Figure 4, What is the host gene used to normalize the Wolbachia copy? This information should be included in method. There is no PNG (the highest one) density in Fig.4A. Statistic information should be provided in the figure.

**The host gene is *Ae. albopictus* ITS2 gene. We have described the details of these primers (line190-196). We have now included PNG in both Fig 4A and Fig 4B. Statistical information has been provided in the figure and updated.**

2. Why not validate the sex through assay of Nix gene by PCR? Is this more straightforward and easier than ddRAD-seq?

**We agree that screening Nix gene by qPCR would be more straightforward than ddRAD-seq. However, this is likely a misunderstanding caused by our descriptions in method section. We have rewritten these parts for clarity (line 103 and line 135). The ddRAD sequencing data we used for sex classification in *Ae. albopictus* samples was obtained from our previous study, which focused on population genetic analysis and has been published. For the present study, we revisited those data and used the ddRAD-seq data for sex and *Wolbachia* classification.**

3. The age of sample can be a big factor affecting the variation of Wolbachia infection between sexes as male can lose wAlbA with aging. This probably is more important reason than others in term of higher rate of wAlbB in males than females in the tested samples.

**We certainly appreciate this point and had already mentioned it in relation to density. However it is not clear that survival rates of *albopictus* differ much between males and females and some studies show similar estimates (e.g. Lacroix et al 2009). We consider possible infection loss effects at lines 478-480 and have altered wording around some age effects at lines 499-501.**

**R. Lacroix, H. Delatte, T. Hue, P. Reiter, Dispersal and Survival of Male and Female *Aedes albopictus* (Diptera: Culicidae) on Réunion Island, *Journal of Medical Entomology*, Volume 46, Issue 5, 1 September 2009, Pages 1117–1124,** [**https://doi.org/10.1603/033.046.0519**](https://doi.org/10.1603/033.046.0519)
